# Effects of Bromelain and Papain in Tooth Whitening and Caries Removal: A Literature Review

**DOI:** 10.3390/dj13030132

**Published:** 2025-03-16

**Authors:** Stanca Cuc, Amalia Moldovan, Marioara Moldovan, Codruta Sarosi, Smaranda Buduru, Cecilia Bacali, Doina Prodan, Viorica Lazar, Sorin Claudiu Man

**Affiliations:** 1 Department of Polymeric Composites, “Raluca Ripan” Institute of Chemistry, Babeș-Bolyai University, 400294 Cluj-Napoca, Romania; stanca.boboia@ubbcluj.ro (S.C.); marioara.moldovan@ubbcluj.ro (M.M.); liana.sarosi@ubbcluj.ro (C.S.); doina.prodan@ubbcluj.ro (D.P.); 2 Physics and Chemistry Department, Technical University of Cluj-Napoca, 28 Memorandumului Str., 400114 Cluj-Napoca, Romania; amalia.mazilu@gmail.com; 3 Department of Prosthodontics and Dental Materials, “Iuliu Hatieganu” University of Medicine and Pharmacy, 400006 Cluj-Napoca, Romania; smarandabuduru@yahoo.com; 4 Department of General Medicine, Vasile Goldis University of Medicine, 310048 Arad, Romania; docleordean@yahoo.com; 5 Pediatric Clinic II, Clinical Hospital Emergency of Arad County, 310037 Arad, Romania; 6 Department Paediat 3, Mother & Child Department, “Iuliu Hatieganu” University of Medicine and Pharmacy, 2-4 Campeni Str., 400217 Cluj-Napoca, Romania; sorinman@gmail.com

**Keywords:** proteolytic enzymes, whitening gels, hydrogen peroxide, anti-inflammatory

## Abstract

**Background/Objectives:** The objective of this review is to establish a solid base of information regarding the use of proteolytic enzymes to replace hydrogen peroxide/carbamide in teeth whitening products. The use of proteolytic enzymes, such as bromelain and papain, can provide surprising results for solving two important aspects related to dental aesthetics: tooth whitening and the chemo-mechanical removal of damaged dental tissue. Due to their ability to degrade salivary proteins, these enzymes can be used successfully as active agents in tooth whitening and in the atraumatic treatment of caries without being accompanied by other side effects on dental components. **Methods:** Random-effects meta-analyses were performed with enzymes (bromelain, papain) used in dentistry. A keyword search of scientific publications was conducted using the Google Academic, Web of Science and PubMed search engines. **Results**: The results were systematized in the present work in two parts: bromelain and papain effects in tooth whitening and chemo-mechanical/atraumatic removal of damaged dental tissues. **Conclusions:** The findings from different studies and clinical reports indicate that bromelain and papain could be considered efficient and safe therapeutic agents not only in various medical conditions but also in dental problems.

## 1. Introduction

In modern dentistry, there is a continuous search for efficient techniques that are less invasive, are less harmful and have minimum discomfort for patients. In the same line of thought, dental materials with improved physical and chemical properties, with high biocompatibility and without harmful effects on dental tissues have been investigated. Bromelain and papain are natural substances that have shown great potential in systemic diseases as well as in dental treatments. Tooth whitening and atraumatic treatment of caries are dental therapies that can benefit from the use of natural substances like proteolytic enzymes.

In traditional teeth whitening, the effect of whitening agents is mainly associated with the abrasive effect on extrinsic stains [1,2]. However, the common substances used to improve tooth color can have harmful effects on dental structures, which has led to the search for safer alternatives [3,4]. Proteolytic enzymes, such as bromelain and papain, have shown potential in teeth whitening in in vitro studies [5,6] due to their ability to enhance the catalytic oxidation of peroxidase and reduce the toxicity of electron donors such as peroxides [7,8]. Dental products with bromelain and papain are as effective as commercial ones in removing stains [9] and less abrasive to dental tissues [2].

The whitening mechanism is based on the ability of these enzymes to break down the organic compounds that cause the formation of extrinsic stains on the tooth’s surface. Proteolytic enzymes can break peptide bonds in proteins. The proteins in the pellicle (salivary film) adhere to the surface of the enamel and are the main components that retain the colored pigments from food, drink or tobacco. The action of these enzymes degrades the protein layer, reducing the stain-retention capacity [2].

Bromelain has proteolytic activity and can break down the complex oxidized protein molecules that form the extrinsic spots. Papain, in addition to its proteolytic activity, also has the effect of reducing oxidized compounds by catalyzing chemical reactions that break down organic pigments.

Enzymes help increase the efficiency of peroxidase, a chemical process that oxidizes colored pigments, turning them into less colored or colorless compounds. By reducing the toxicity of electron donors such as peroxides (often used in conventional whitening), papain and bromelain act as safer and less aggressive alternatives to protect the enamel [2].

**Figure 1:**
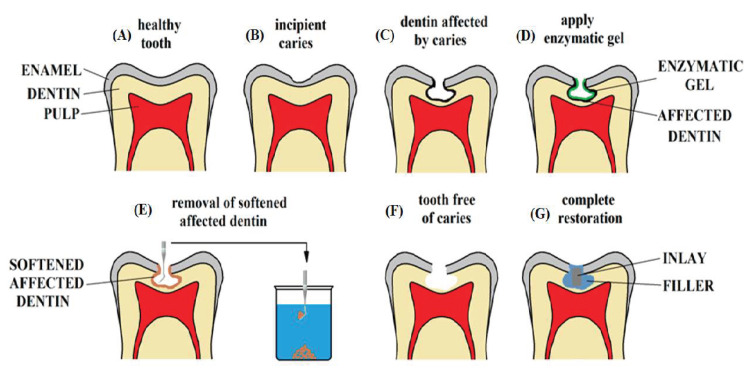
Scheme of atraumatic treatment of caries using enzymatic gels.

In dentistry, the use of papain gel in atraumatic treatment of caries has shown antimicrobial, bacteriostatic and anti-inflammatory properties together with biocompatibility, being able to accelerate the healing process once the affected tooth tissue is removed from dental caries [10]. Figure 1 shows the scheme of atraumatic treatment of caries with enzymatic gels.

Chemo-mechanical techniques based on enzymes, such as bromelain and papain, allow for the selective degradation of infected dentin, preserving healthy tooth structure. These enzymes break down collagen fibrils in carious dentin, soften the affected tissue and facilitate removal with minimal mechanical force [10]. They also contribute to the breakdown of bacterial biofilms, eliminating bacteria and preparing the cavity. Unlike traditional drilling methods, the use of enzymes minimizes the loss of healthy dentin, reducing the need for extensive restorations. The anti-inflammatory properties of bromelain and papain promote faster healing after treatment. These medical and industrial enzymes have been widely applied for their beneficial effects and uses.

Bromelain is a proteolytic enzyme from the group of cysteine-proteinases, capable of breaking down proteins into amino acids [11,12]. Chemically discovered in 1876 [13] and identified in 1891 [14], it contains proteolytic sulfhydryls, peroxidases, phosphatases, protease inhibitors and organically bound calcium [15,16]. It is present in the stem and fruits of pineapple [17] but also in the waste resulting from its processing [18,19]. There are two types, stem bromelain (SBM) and fruit bromelain (FBM), which differ in structure and properties [17,20,21,22].

Pineapple (*Ananas comosus*) is one of the most consumed fruits worldwide, cultivated in tropical and subtropical regions [23,24]. Bromelain is used in the food industry [25,26], in breweries [27,28] as well as in the textile and cosmetic industries [29,30]. Its optimal activity takes place in a pH range of 5.5–8.0 and at a temperature of 50–70 °C [31,32].

Due to its varied clinical applications, bromelain has been intensively studied for its anti-inflammatory, anti-edematous, anti-thrombotic and fibrinolytic effects [25,33,34,35]. However, the presence of impurities decreases its enzymatic activity, which is why research has focused on simple purification methods [16,36,37].

Papain is another proteolytic enzyme, extracted from the latex of papaya (Carica papaya) leaves and fruits; papaya is an exotic fruit rich in nutrients, fiber, vitamins and antioxidants [38,39]. Used in the food and pharmaceutical industries [40], papain is available as a supplement, as a topical application, or in combination with bromelain. It has antibacterial [41], anti-inflammatory [42], antioxidant [43], antiviral [44,45,46], antitumor [47] and cicatrizing [48] properties.

These enzymes present a significant potential in the medical and industrial fields due to their beneficial effects and widespread use.

## 2. Materials and Methods

The objective of this review article was to investigate the effect of bromelain and papain in tooth whitening and caries removal. A systematic search was performed in scientific databases, including PubMed, Web of Science and Google Scholar, using the following keywords: <bromelain and/or papain in teeth whitening>, <enzyme in caries removal>, <peroxide carbamide and/or enzyme in teeth whitening>.

This search aimed to gather relevant articles that would allow for a comprehensive review of the topic.

Inclusion Criteria. The following criteria were applied to ensure the inclusion of high-quality, relevant studies:

Publication date: Studies published within the last 10 years were included to ensure the research is current;

Full-text availability: Only articles that provided full-text access were considered to ensure the detailed methodology and results could be fully reviewed;

Language: Articles published in English were included to avoid potential issues with translation or understanding.

Exclusion criteria. The following types of articles were excluded:

Articles available only as abstracts, as these do not provide sufficient detail for evaluation;

News articles, letters, interviews and opinion pieces, as these do not offer empirical research data;

Non-English texts, as these may present language barriers.

Screening Process. The screening process consisted of two stages. Stage 1: Title and Abstract Screening. Initially, the titles and abstracts of the identified articles were reviewed. Only those that directly related to tooth whitening or the chemo-mechanical removal of damaged dental tissues, specifically in relation to microorganisms in the oral cavity, were selected for further analysis.

Stage 2: Full-Text Screening. After the initial selection, a detailed review of the full-text articles was conducted to ensure they met all the eligibility criteria. This stage provided a deeper understanding of the methodologies, results and relevance of each study.

Data Structuring. The results of the included studies were organized into two primary categories for presentation: Effects of Bromelain and Papain in Tooth Whitening; Chemo-Mechanical/Atraumatic Removal of Damaged Dental Tissues.

**Figure 2:**
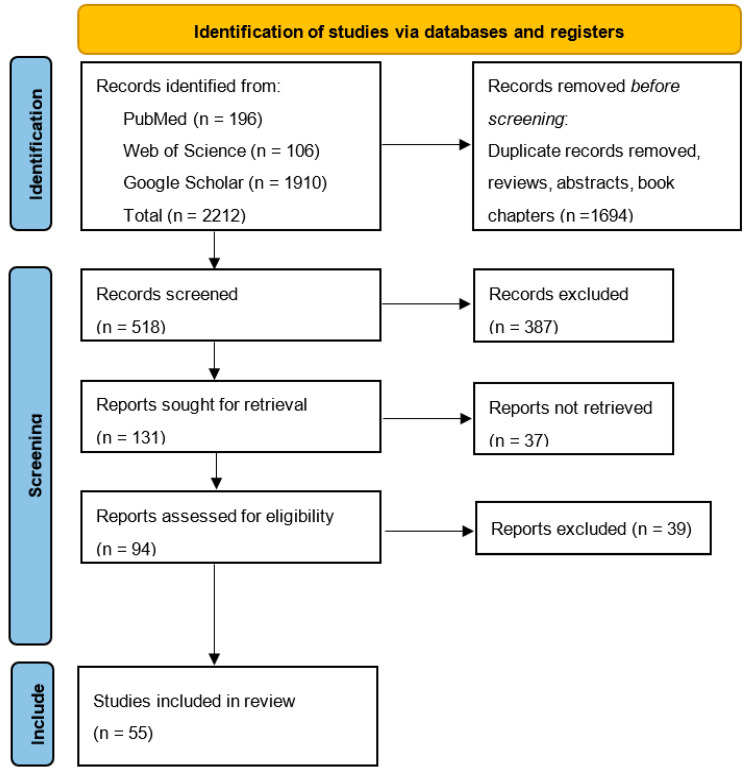
PRISMA flow diagram.

Quality Assessment. To assess the methodological quality of the included studies, standardized tools such as PRISMA (Preferred Reporting Items for Systematic Reviews and Meta-Analyses) were applied. This ensured that the review adhered to established guidelines for systematic reviews and provided reliable and unbiased conclusions (Figure 2).

## 3. Results and Discussion

### 3.1. Bromelain and Papain Effects in Tooth Whitening

Tooth whitening is a treatment that is highly requested by patients in the dental office, as a nice smile is generally considered an expression of self-confidence, happiness and oral health. The effect of bromelain and papain used for improving tooth color was evaluated in many recent studies in the attempt to find natural, mild solutions that can be used in dental treatments as alternatives to other more irritative materials. The effects of bromelain and papain in tooth whitening compared to hydrogen peroxide agents are presented in Table 1.

The hydrogen peroxide solution that is usually used in tooth whitening has several side effects on the dental and oral tissues: it permanently alters enamel structure [49] by organic chromophore oxidation [50], degrades the organic matrix and decreases the microhardness value of the tooth [49]; hypersensitivity can often occur as a result of hydrogen peroxide pulp irritation by penetration in the exposed dentinal tubules [50]. Erosion was also found after tooth bleaching [51] as well as decreased cell viability [41].

Among the oral effects of bromelain are degradation of tooth staining proteins, tooth color improvement and a brighter smile by a better light reflection [50], preservation of fibroblast integrity after teeth bleaching [50], anti-inflammatory response [12], low levels of cytotoxicity and less discomfort for the patient than that caused by peroxide solutions. The use of bromelain can help in systemic diseases, like chronic inflammation, arthritis, autoimmune diseases and tumors [12]. Anti-thrombotic and fibrinolytic effects [50], complete burn debridement [24], biofilm reduction and gingivitis improvement are also associated with bromelain [55]. In a review on the use of bromelain in tooth whitening and the comparison between bromelain and peroxide effects, studies showed that, while hydrogen peroxide has several adverse effects on tooth structures, bromelain is a natural and accessible product that can be considered an alternative therapeutic agent in tooth bleaching [3].

The mechanism and effectiveness of hydrogen peroxide whitening with or without natural additives were discussed in different in vitro and in vivo studies by analyzing tooth characteristics such as crystallinity index, shade, lightness and chroma [53,54,58].

Eimar et al. analyzed the mechanism of tooth whitening when using hydrogen peroxide solutions and found that the improvement in tooth color is due to the organic matrix oxidation. Changes in enamel component ratios, crystallinity index and tooth lightness were determined in four groups of sound teeth treated respectively with NaOH solution that deproteinizes the organic content, EDTA solution that decalcifies the mineral content, oxidizing H_2_O_2_ solution and distilled water as a control. The highest increase in tooth lightness was obtained when hydrogen peroxide was used by oxidation of the tooth organic matrix without significant changes in enamel elemental ratios [53].

Vekaash et al. investigated the effect of the addition of pineapple extract to hydrogen peroxide solutions of different concentrations (30%, 20% and 10%) for bleaching artificially stained teeth. The two halves of each tooth were treated with the hydrogen peroxide solution with and without the addition of pineapple extract. The effectiveness of the association between peroxide and pineapple extract was also compared with that of a peroxide solution using spectrophotometer reflectance to record the tooth color variables according to the CIEL*a*b color system. Higher color values were obtained when using the natural additive, showing higher bleaching efficacy when the pineapple extract was used [58].

Other authors also evaluated the efficacy of tooth whitening formulations and dentifrices with or without papain and bromelain addition [61,62].

Stain removal efficacy of a dentifrice containing proteolytic enzymes (papain and bromelain extracts) compared to a commercial dentifrice was evaluated by analyzing the luminosity values using pre- and post-treatment photographs. The addition of proteolytic enzymes to a dentifrice formulation showed an increased effect on stain removal [61].

Experimental whitening formulations (containing papain, ficin and bromelain) were compared with carbamide peroxide regarding the effects on tooth color, roughness, microhardness and cell viability. Forty bovine dental discs previously stained were treated with the four studied gels, and then, measurements were performed. While carbamide peroxide produced a decrease in enamel hardness and an increase in enamel roughness, the other gels showed similar color changes. The substantial clinical potential of peroxide-free whitening gels was the result [50].

In another study, Munchow et al. compared the color changes obtained after a papain-based gel, bromelain-based gel, carbamide peroxide solution, and also a no-agent solution (control) were used for 4 weeks for bleaching bovine teeth (stained for a week in coffee solution). The color changes were measured with a digital spectrophotometer using the CIEL*a*b* color system. All the stain removal gels showed color changes that were more evident after the second application. Promising results for the proteolytic enzymes used in tooth whitening were obtained [62].

The whitening effect of a dentifrice containing two proteolytic enzymes (papain and bromelain) was tested by Ananthakrishna et al. in a clinical study on 35 patients who brushed their teeth twice daily during a 3-week period. The examination of the patients after one, two and three weeks using a Vita shade guide showed significant improvement of the tooth color [63].

The mechanism of tooth discoloration produced after papain addition (in different concentrations) to saliva was investigated by Yoshikawa et al. Salivary proteins were also analyzed by Coomassie Brilliant Blue staining, resulting in a large degree of protein degradation. The proteins absorbed onto a hydroxyapatite powder that was added to saliva in the other samples were also degraded. Hence, the papain capacity to improve (in a concentration-dependent manner) tooth color by degrading the salivary proteins bounded to the stains and by decomposing the surface pellicle was demonstrated [64].

Solanki et al. [59] compared the effectiveness of hydrogen peroxide combined with pineapple and papaya extracts for teeth whitening. Their findings showed that the pairing of hydrogen peroxide with bromelain from pineapple had the highest efficacy, followed by the combination with papaya.

Mazilu et al. described the preparation and then evaluated the antibacterial properties of five natural whitening gels containing bromelain, quince and whey in different proportions. Two other gels containing Lubrizol and SiO_2_ were prepared. Five bacterial strains were used in the study: *Streptoccocus mutans*, *Porphyromonas gingivalis*, *Enterococcus fecalis*, *Escherichia coli* and *Staphyylococcus aureus.* In vitro analyses based on SDS-PAGE, UV–VIS, FTIR spectroscopy and SEM microscopy were performed. The results showed antibacterial effects of all the gels except the one containing SiO_2_. Different diameters of inhibition were obtained when testing different microbes [56].

**Figure 3:**
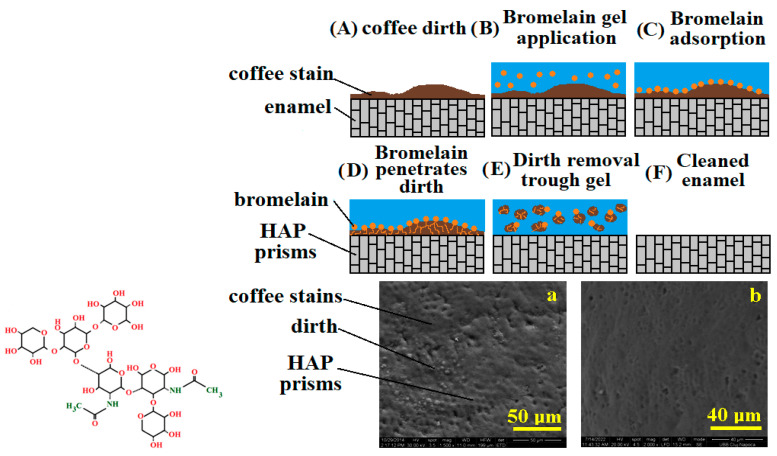
Bromelain effect in bleaching gel against coffee stains. SEM image of dental enmamel treated with experimental gel with bromelain at different magnification (**a**) 50 μm and (**b**) 40 μm.

In Figure 3, you can see the chemical formula of bromelain and the stages of action of this enzyme on coffee stains on tooth enamel (application of the gel with bromelain, adsorption of bromelain on the coffee stain, penetration and fragmentation of dirt deposits on the enamel and, finally, cleaning). You can also see the SEM images before and after cleaning of the enamel stained with coffee.

**Figure 4:**
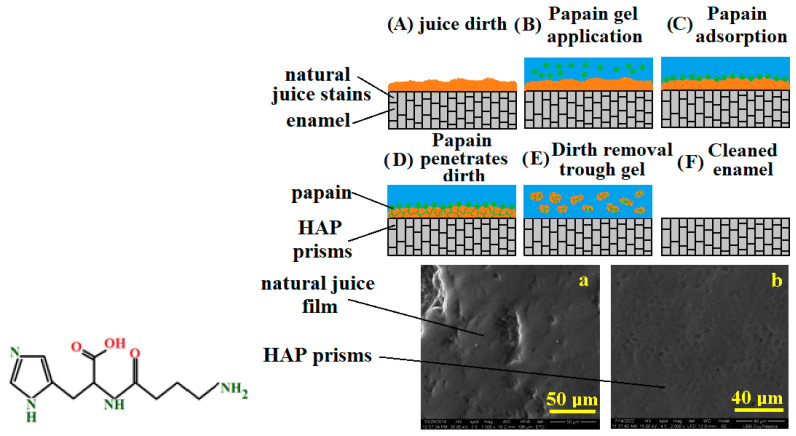
Papain effect in bleaching gel against natural juice stains. SEM image of dental enmamel treated with experimental gel with papain at different magnification (**a**) 50 μm and (**b**) 40 μm.

In Figure 4, you can see the chemical formula of papain and the stages of action of this enzyme on juice stains on tooth enamel (application of the gel with papain, adsorption of papain on the juice stain, penetration and fragmentation of dirt deposits on the enamel and, finally, cleaning). You can also see the SEM images before and after cleaning of the enamel stained with juice.

Chhabile et al. showed that the attachment of certain bacteria associated with dental plaque, gum health or periodontal diseases can be selectively affected by the higher amounts of specific enzymes (like papain, trypsin and chemotrypsin) by modifying the cells’ receptivity. A similar phenomenon is found in the composition of gingival flora that change as a consequence of poor oral hygiene, favoring gingival diseases. Epithelial cell treatment with enzymes has been shown to have an influence on bacterial attachment and colonization on certain tissues. It reduced the number and attachment of *Streptococcus sangvis* and *Streptococcus mitis* (strains associated with periodontal diseases). By contrast, a higher number of *Bacteroides gingivalis* and *Bacteroides intermedius* (strains associated with destructive periodontal diseases) were found. The number of *Actinomyces viscosus* (species associated with dental plaque) increased after cell treatment with neuramidase and decreased after cell treatment with protease [66].

### 3.2. Chemo-Mechanical/Atraumatic Removal of the Damaged Dental Tissues

Dental caries is an important oral health problem all around the world that affects most of the children and the majority of adults, mainly caused by poor oral hygiene and high carbohydrate consumption [67,68,69,70].

The first manifestation of the decomposition process is a small area of demineralized enamel on the tooth surface, often hidden from sight in tooth cracks or between interproximal surfaces. Next, the destruction extends to the softer dental tissues, the loose enamel collapses to form a cavity, and the tooth is progressively destroyed. Caries usually affects the tooth crown but can also affect the tooth root if the dentin is exposed as a consequence of gum recession; this situation is more common in older adults [71,72,73,74].

Dental caries is caused by the action of acids (released by cariogenic bacteria from the plaque) on the enamel surface of both temporary and permanent teeth. Acid is produced when sugars (mainly sucrose) from foods or drinks interact with bacteria from the dental plaque present on the tooth surface, producing a loss of calcium and phosphate from the enamel through a demineralization process [75,76,77,78]. The treatment of a dental cavity includes several specific steps. The dentist first drills the tooth in the affected area, then removes the damaged tissues and finally places a restoration (filling, inlay) using different types of materials, such as composite resins, glass ionomers, ceramics, etc. Without treatment, the small caries would evolve into extended dental caries that may require a crown, a treatment of the root canal or even an implant after tooth extraction.

Alternative procedures, like air abrasion [79,80], sonic abrasion, ultrasonic methods and lasers [81,82], were also proposed for caries treatment in order to avoid the pain, discomfort and fear associated with conventional treatment [83].

Since 1975, a less invasive alternative to the traditional treatment—the atraumatic restorative treatment (ART), based on the chemo-mechanical removal of caries—has been introduced. Many in vitro and in vivo studies have been carried out on this topic, and their results are presented in Table 2 (Study/Results).

In an article on Carisolv, Kathuria et al. discussed the mechanism of action, advantages, disadvantages, indications and contraindications of this chemo-mechanical, conservative treatment that can be associated or not with the traditional method. The atraumatic treatment of caries enables the preservation of healthy tissue, while the altered tissue is removed; in addition, it is more comfortable for the patients [84].

In another review on the chemo-mechanical removal of caries based on in vitro and in vivo studies, Hamama et al. analyzed the different methods and agents (based on enzymes or based on sodium hypochlorite) used in the atraumatic treatment of caries as well as their mechanism of action, working time, effects on dental tissues, historical evolution and the patients’ perception. The particularities and efficacy of sodium hypochlorite-based treatment using Caridex and Carisolv as well as enzyme-based methods using products like Papacarie or Biosolv compared to rotary instrumentation were discussed. The results showed that these methods could be preferred in the case of patients who are stressed by the conventional treatment, especially children and uncooperating and anxious patients [85].

Papacarie, a papain-based gel, showed efficacy in the chemo-mechanical removal of infected dentine, being an easy to manipulate and cheap material that is applied using a simple procedure [86].

The efficacy on caries removal of two chemo-mechanical agents, Carisolv and Papacarie, was studied by Kumar et al. on forty patients with Black class I dentinal caries. The time needed for caries removal as well as the mean volume of affected tissue removal were evaluated in the study. The volume of the resulting cavities was assessed by a microscopic analysis of the casts obtained after pouring the silicone impression of the studied teeth. Although Papacarie exhibited slightly better effects, both gels showed overall similar activity on caries removal [90].

Mancini et al. used a papain-based gel to treat an interproximal cavity by applying the BRIX3000 gel directly in the cavity for 2.5–3 min and then removing the infected dental tissue in three steps. No discomfort was reported by a patient who was previously frightened of the mechanical treatment that used a turbine. The gel had a chemical effect on bacteria and modified the consistency of the altered dental tissues, proving to be a good alternative to the use of a turbine and a less traumatic, less painful treatment of dental caries [87].

Pain and discomfort among children evaluated during two different caries removal treatments (Papacarie and Atraumatic Restorative Treatment ART) were studied by Khaleh et al. in a randomized, controlled and blinded clinical trial including fifty patients. The treatment evaluation was performed by two independent investigators who used the Sound, Eye and Motor Scale (SEM) and also measured the working time. The treatment using Papacarie gel required a longer time for altered tissue removal compared to ART. However, the pain and discomfort were reduced when using Papacarie compared to ART. Regression analysis was used to evaluate the results [97].

The effect of Papacarie gel on deciduous teeth was also investigated by Bussadori Leal de Godoy et al. Papacarie gel was used for chemo-mechanical caries removal, followed by the infected dentin removal with a blunt curette and then the restoration of the cavities using glass-ionomer on 84 deciduous posterior teeth with occlusal dentinal caries without risk of pulp exposure. At the radiographic evaluation, 12 months after the restorations were performed, resorption and calcification of the affected teeth were noted. The difference between the treatment success and treatment failure was significantly different, with a success rate of 88.1% based on the clinical evaluations and 98.8% for the radiographic evaluations. The study proved the efficacy of Papacarie gel on deciduous teeth [93].

In a study that compared the efficacy of a chemo-mechanical caries removal agent and a conventional method of caries removal, Jawa et al. found a reduced destruction of dentinal tubules when using atraumatic methods. The effects of Papacarie, a papain-based gel used as a chemo-mechanical agent for caries removal, was compared with those of a slow-speed rotary instrument. Twenty extracted human teeth with extensive carious lesions were used in the study; the removal of the caries was performed using traditional methods, with slow-speed rotary burs, and also a chemo-mechanical method using Papacarie, a therapeutic agent based on the bactericidal, bacteriostatic and anti-inflammatory effects demonstrated by papain. The samples from the treated teeth underwent a microscopic examination in order to check for the presence of bacteria and the aspect of the dentinal tubules. Though an efficient removal of destructed dental tissue was obtained for both methods, Papacarie was a good alternative to the classical treatment, a more conservative and comfortable procedure, less traumatic on dentinal tubules and less invasive and can be highly useful especially in pediatric dentistry [98].

Silva et al. analyzed the action on the sound collagen microstructure of a papain-based gel, Papacarie (TM), used for an atraumatic treatment of caries by chemo-mechanical removal of the affected dental tissue. The adsorption of the Papacarie gel as well as the integrity and microstructure of type I collagen from bovine Achilles deep tendon were evaluated on 10 samples using the attenuated total reflectance technique of Fourier transform infrared (ATR-FTIR). The results showed that the studied gel does not affect the collagen structure, indicating that the dentine is capable of remineralization. It was thus demonstrated that, by using the papain-based gel, a selective removal of the affected dentine is possible [94].

Coirêa et al. evaluated the microhardness of the remaining dentin following caries removal in primary teeth using two different techniques: conventional using a low-speed bur and chemical using two different products. Although a reduced hardness of the remaining tissues was found when using chemo-mechanical methods (Papacarie and Carisolv), there was no statistical significance between these treatments of caries [95].

The effect of an enzymatic treatment with papain gel on the structure of intact and nonmineralized type I collagen fibers from the dentin matrix was evaluated by Bertassoni. Partial degradation of the surface with no rupture of the fibers was noticed on atomic force microscopy (AFM) images. A gradual decrease in the elastic modulus and hardness of the healthy dentin that can be a consequence of the degradation of matrix proteoglycans was obtained after four applications (of 30 s each) of the papain gel [88].

Lopes et al. evaluated the effect of a chemo-mechanical caries treatment that used a papain-based gel on the shear bond strength of a restorative material. Healthy and demineralized dentin samples were used in the study. After being treated with the papain gel, the samples were stored in a damp environment for 7 days, and then, the fracture mode was analyzed using a universal testing machine. Adhesive and cohesive fractures of the resin were found in all the samples, with no differences in the shear bond strength values, showing that the use of the papain-based gel did not affect the bond strength of the adhesive restorative system [89].

The inhibitory effect on calculus formation of a toothpaste containing papain and pyrophosphate was evaluated by Ardani et al. Measurements of the levels of salivary calcium, phosphate and pH were performed on unstimulated saliva using an atomic absorption spectrophotometer (SAA) and a pH meter, respectively. Decreased calcium and phosphate levels were obtained, showing an anti-plaque effect of the experimental toothpaste, by a lower calcium phosphate precipitation on the enamel. The salivary pH, maintained normal after the enzymatic treatment, supports the inhibitory effect of the studied toothpaste on dental plaque. The results were due to the antibacterial and anti-inflammatory effect of papain and also the pyrophosphate antimicrobial and stabilizing role on phosphate levels that inhibit the formation of calculus. The use of the toothpaste formulation containing pyrophosphate and papain could help in reducing dental plaque and calculus accumulation in patients with fixed orthodontic appliances [99].

Bromelain anti-inflammatory effects on human dental pulp cells, demonstrated by Hong et al., open the possibility for bromelain to be used in vital pulp treatment. Decreased NF-kB and MAPK pathways with decreased p65 phosphorylation levels in the cytoplasm and nucleus, reduced inhibition of inflammatory cytokine expression (decreased phosphorylation levels of extracellular signal-related kinases/p38 mitogen-activated protein kinases) as well as preserved cell viability were obtained after exposing the cells to a solution containing lipopolysaccharide and bromelain [100].

Bromelain was found to be more efficient than papain in the chemo-mechanical removal of caries in a study performed by Reddy et al. using organic gels. Thirty molars with caries that affect the dentine were treated with bromelain or papain; then, the time needed for caries removal and the amount of the residual demineralized dentin were measured and analyzed using a stopwatch, a stereomicroscope and a weld check software. While the working time was almost similar, the layer of remaining demineralized dentin was thinner when using bromelain than when using papain [101].

Bromelain activity was discussed by Mameli et al. [102] in an overview of its applications in medicine and dentistry. Several effects of bromelain were found when used in systemic disease treatments: decreased platelet aggregation; reduced edema, inflammation, and pain; antimicrobial action; antitumoral activity; as well as low toxicity and low side effects, though gastrointestinal and allergic reactions were mentioned by different studies. In dentistry, as well, many bromelain effects, like reduced inflammation and edema, prevention of caries and also antibacterial activity, were investigated [96].

Unlike the chemical and mechanical agents used in traditional whitening, products with papain and bromelain offer a gentler effect on enamel and dentin, minimizing abrasion. Enzymes do not affect the mineral structures of enamel and dentin, making them a safe alternative [1].

Bromelain and papain are both proteases, meaning they function by hydrolyzing peptide bonds in protein-based stains and plaque biofilms. Stains on the surface of dental enamel are caused by pigmented compounds that adhere to the enamel through proteinaceous films. By breaking down these films, the enzymes facilitate the removal of extrinsic stains, increasing light reflection from the tooth surface and resulting in a whitening effect [62,65].

In comparison to hydrogen peroxide/carbamide-based whitening products, which act by generating reactive oxygen species known to alter the structural and biochemical properties of hard tissues and dental pulp through significant corrosion of the enamel’s nanostructure, enzymes operate through a distinct mechanism without producing reactive oxygen species. Bromelain and papain act enzymatically, reducing enamel erosion and preserving tooth integrity, offering a gentler alternative with lower risks of irritation [64].

The development of dental caries occurs due to bacterial biofilms composed of microorganisms embedded in an extracellular polymeric matrix. Bromelain and papain can degrade this matrix, weakening bacterial adhesion and facilitating the removal of harmful microbes through mechanical cleaning [53]. Both enzymes exhibit antimicrobial activity, reducing bacterial load while also having anti-inflammatory effects that may help soothe gum irritation. As naturally derived enzymes, bromelain and papain are considered safe for dental applications, making them suitable for individuals with sensitivity [65].

## 4. Conclusions

The findings from different studies and clinical reports indicate that bromelain and papain could be considered efficient and safe therapeutic agents not only in various medical conditions but also in dental problems. Experimental gels containing enzymes of plant origin as active agents may have significant clinical potential for the preparation of gels free of hydrogen/carbamide peroxide, substances that can affect the dental tissues and cause sensitivity due to their migration into the dentinal tubules. Proteolytic enzymes like bromelain and papain can be used in tooth bleaching because of their capacity to degrade the salivary proteins bound to the stains due to their role in breaking proteins into smaller components. Atraumatic treatment of caries can also benefit from the use of bromelain and papain, as they are able to dissolve the tooth inorganic component and soften the altered tissues without side effects on the dental components. For their dental use, the efficacy and mechanisms of actions of proteolytic enzymes must be further tested. Additional clinical studies that evaluate the effects of different papain and bromelain concentrations are recommended to determine the in vitro/in vivo dose–response relationship.

## Figures and Tables

**Table 1:** Studies about bromelain and papain effects compared to hydrogen peroxide agents in tooth whitening.

Articles	Results	References
Vilhena K.F.B. et al. Ribeiro J.S. et al. Müller-Heupt L. K. et al. Lilaj B. et al. Al-Badri H. et al. Barbosa L.M. et al.	Hydrogen peroxide side effects: - organic chromophore oxidation - decreased enamel hardness - increased enamel roughness - hypersensitivity due to penetration in the exposed dentinal tubules - decreased tooth microhardness - erosion - decreased cell viability - organic matrix oxidation, increased tooth lightness	[49,50,51,52,53,54]
Medina-Zamora N. et al. Mohamed Thameemul Ansari K.A. et al. Hikisz, P. Ribeiro J.S. et al. Jurema A.L.B. et al. Mazilu A. et al. Parlis Y. et al.	Bromelain effects: - proved to be an alternative therapeutic agent in tooth bleaching - complete burn debridement - anti-inflammatory response - effects in systemic diseases like chronic - inflammation - arthritis - autoimmune diseases and tumors - degradation of staining proteins/brighter smile - preservation of fibroblast integrity - anti-thrombotic and fibrinolytic effects - biofilm reduction and gingivitis improvement - antibacterial effect	[3,24,34,50,55,56,57]
Vekaash C. et al.	Hydrogen peroxide + bromelain (different concentrations): - higher bleaching efficacy	[58]
Solanki M.N. et al. Mazilu M.A. et al.	Hydrogen peroxide + papain: - significant bleaching efficacity	[59,60]
Patil P. A. et al. Shobana G. et al. Munchow E. et al. Ananthakrishna S. et al.	Bromelain + Papain toothpaste effects: - efficient stain removal - improved lightness values - increased effect on stain removal - improved luminosity values - improvement of tooth color - stain removal - promising results in tooth whitening	[5,61,62,63]
Yoshikawa Y. et al. Chakravarthy P.K. et al. Chhabile S. et al.	Papain effects: - improvement of tooth color by degradation of staining proteins bonded to the teeth - reduced bacterial attachment and - colonization	[64,65,66]

**Table 2:** Studies about the atraumatic restorative treatment (ART).

Articles	Results	References
Kathuria V. et al.	Atraumatic treatment: - Carisolv removed altered tooth tissues - Carisolv preserved sound tissues, the treatment being comfortable for the patient	[84]
Hamama H. et al.	Chemo-mechanical methods using sodium hypochlorite-based gels (Caridex and Carisolv) and enzyme-based gels could be used to reduce the treatment stress in caries therapy.	[85]
Kulkarni G. et al. Mancini L. et al. Bertassoni L.E. et al. Lopes M.C. et al.	Papacarie (Papain gel): - efficient infected dentine removal, easy to manipulate, cheap - used in interproximal cavity: short treatment time, modified consistency of the dentine, no discomfort reported, less pain - determined partial degradation of the surface but no rupture of the fibers; a gradual decrease in elastic modulus and hardness of the healthy dentin after several applications was also found - did not affect the bond strength of the adhesive restorative system	[86,87,88,89]
Kumar J. et al.	Carisolv and Papacarie: - showed similar treatment time and volume of removed affected tissue in Black class I dentinal caries	[90]
AlHumaid J. et al. Chittem J. et al. Bussadori S.K. et al. Silva Z.S. Jr et al. Coirêa F.N.P. et al. Ozsoy S. et al.	Papacarie gel: - efficacy demonstrated in permanent teeth caries - softening of altered tissue obtained - efficient, easy and comfortable when used in treatment of caries in adolescent patients by dissolving the minerals in exposed collagen and softening the dentine - efficacy on deciduous teeth - does not affect the collagen structure, permits a selective removal of affected dentine - the product is safe to use in caries treatment - determined a reduced hardness of the remaining tissues, but the effect was similar to the conventional technique	[91,92,93,94,95,96]
Khaleh A.A. Jawa D. et al.	Papacarie: - reduces pain and discomfort in caries treatment of children when compared to ART - exhibits a less invasive action, a reduced destruction of dentinal tubules, then a slow-speed rotary instrument	[97,98]
Ardani I.G.A.W. et al.	Papain toothpaste: - inhibition effect on calculus formation, antibacterial and anti-inflammatory effect of papain	[99]
Hong J.H. et al. Reddy V.S. et al. Maneli A. et al.	Bromelain: - anti-inflammatory effects on human dental pulp cells, preserved cell viability - more efficient than papain in the chemo-mechanical removal of caries removal, resulting in a thinner layer of demineralized dentin - demonstrated several effects in medicine/systemic diseases (anti-inflammatory, antibacterial, antitumoral, anti-thrombotic, analgesic) and dentistry (anti-inflammatory, antibacterial, caries prevention) together with low toxicity and low side effects	[100,101,102]
